# The Relationship between Fenestrations, Sieve Plates and Rafts in Liver Sinusoidal Endothelial Cells

**DOI:** 10.1371/journal.pone.0046134

**Published:** 2012-09-24

**Authors:** Dmitri Svistounov, Alessandra Warren, Gregory P. McNerney, Dylan M. Owen, Dusan Zencak, Svetlana N. Zykova, Harry Crane, Thomas Huser, Ronald J. Quinn, Bård Smedsrød, David G. Le Couteur, Victoria C. Cogger

**Affiliations:** 1 Centre for Education and Research on Ageing and ANZAC Medical Research Institute, University of Sydney and Concord Hospital, Sydney, Australia; 2 NSF Center for Biophotonics Science and Technology, University of California Davis, Sacramento, California, United States of America; 3 Centre for Vascular Research, University of New South Wales, Sydney, Australia; 4 Eskitis Institute, Griffith University, Brisbane, Australia; 5 Department of Medical Biology, University of Tromso, Tromso, Norway; INSERM U894, France

## Abstract

Fenestrations are transcellular pores in endothelial cells that facilitate transfer of substrates between blood and the extravascular compartment. In order to understand the regulation and formation of fenestrations, the relationship between membrane rafts and fenestrations was investigated in liver sinusoidal endothelial cells where fenestrations are grouped into sieve plates. Three dimensional structured illumination microscopy, scanning electron microscopy, internal reflectance fluorescence microscopy and two-photon fluorescence microscopy were used to study liver sinusoidal endothelial cells isolated from mice. There was an inverse distribution between sieve plates and membrane rafts visualized by structured illumination microscopy and the fluorescent raft stain, Bodipy FL C5 ganglioside GM1. 7-ketocholesterol and/or cytochalasin D increased both fenestrations and lipid-disordered membrane, while Triton X-100 decreased both fenestrations and lipid-disordered membrane. The effects of cytochalasin D on fenestrations were abrogated by co-administration of Triton X-100, suggesting that actin disruption increases fenestrations by its effects on membrane rafts. Vascular endothelial growth factor (VEGF) depleted lipid-ordered membrane and increased fenestrations. The results are consistent with a sieve-raft interaction, where fenestrations form in non-raft lipid-disordered regions of endothelial cells once the membrane-stabilizing effects of actin cytoskeleton and membrane rafts are diminished.

## Introduction

Liver sinusoidal endothelial cells (LSECs) act as a filter between the lumen of the hepatic sinusoid and the surrounding hepatocytes. A major role of the LSEC is to minimize any barrier for the bi-directional transfer of small or soluble substrates between blood and the extracellular space of Disse, while excluding larger circulating particles such as blood cells, platelets and chylomicrons. This physiological role is achieved by the presence of numerous transcellular pores in LSECs called fenestrations. Fenestrations are approximately 50–150 nm in diameter and most are aggregated into groups of 10–100, so-called liver sieve plates [1]. The diameter and number of fenestrations are altered by various liver diseases, diabetes mellitus and old age and are influenced by cytokines and hormones [1]. Alteration in the size and number of fenestrations influences the hepatic trafficking of lipoproteins [2], clearance of pharmaceutical agents [3], liver regeneration [4] and interactions between lymphocytes and hepatocytes [5].

No markers have been reported that specifically label fenestrations and the mechanisms for the regulation of their formation and size remain unclear. The most consistent findings of biological relevance are that fenestrations are increased by actin-disrupting agents [6], [7] and by the angiogenic cytokine, vascular endothelial growth factor (VEGF) [1], [8], [9]. The mechanisms that regulate fenestrations need to be clarified in order to develop strategies to improve lipoprotein metabolism in old age and liver disease [10], and to enhance liver regeneration [4].

Fenestrations are smaller than the limit of resolution of light microscopy and most studies have relied upon electron microscopy with inherent problems related to fixation of tissue. Recently three dimensional structured illumination fluorescence light microscopy (3D-SIM) was applied to LSECs and their fenestrations [11]. 3D-SIM is an ultra-high resolution light microscopy technique that uses interference patterns to convert structures below the resolution limit of light microscopy into observable ones by generating difference/beat frequencies called Moiré fringes. The morphology of the fenestrations and sieve plates was very effectively resolved by 3D-SIM, providing for the first time a detailed three-dimensional map of their structure. Using the plasma cell membrane stain Cell-Mask Orange, discrete membrane structures were identified between the sieve plates. On the basis of their size and appearance we postulated that these structures are membrane rafts and potentially involved in the regulation of sieve plates. Membrane rafts are lipid-ordered domains in cell membranes that vary in size from 10–200 nm, and may aggregate to form micrometer-sized structures [12]. Rafts are enriched in sphingolipids and cholesterol which engenders membrane stability and provides a platform for many membrane proteins such as caveolin. Rafts are tethered to the actin cytoskeleton which has a pivotal role in maintaining their structure and integrity [13], [14]. The size of membrane rafts, like that of fenestrations, is below the limits of resolution of light microscopy and their visualization has mostly been achieved with fluorescence microscopy [15].

In this study, we used 3D-SIM to establish the three-dimensional structure of membrane rafts and liver sieve plates in the cell membranes of LSECs, and the topographical relationship between them. Furthermore, by manipulating membrane rafts and actin, we show how rafts might influence fenestrations, and conclude that rafts are the final regulatory step in the formation of transcellular pores, fenestrations and liver sieve plates.

## Results

### Visualization of Membrane Rafts and Fenestrations

**Figure 1:**
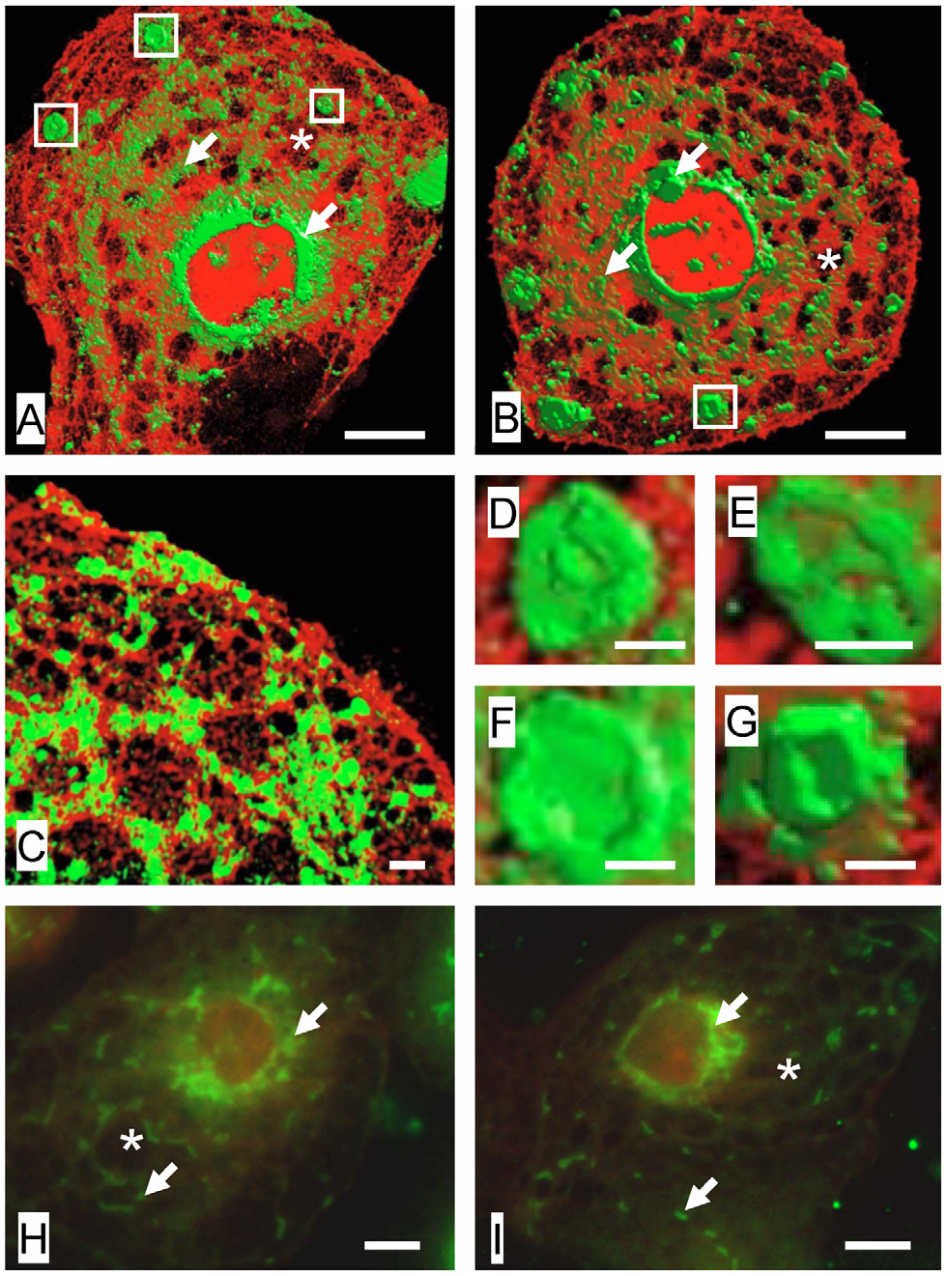
Visualization of membrane rafts and fenestrations. (A–C) 3D-SIM of LSECs stained with Bodipy FL C5 ganglioside GM1, a marker for rafts (green) and Cell-Mask Orange, a cell membrane marker (orange). There is an inverse distribution between liver sieve plates and membrane rafts. Membrane rafts are mostly perinuclear while sieve plates are mostly peripheral. Some sieve plates are identified by an asterix (*) and fenestrations can be resolved within the sieve plates. Rafts are shown with arrows (→). The areas marked in a box (clustered rafts) are further magnified in (D–G). (D–G) Magnification of areas in Figure 1(A–B) showing clustered membrane rafts with raised perimeters. (H) TIRFM of LSEC stained with NBD-cholesterol (green), a marker for rafts, and CellMask Orange (orange) showing perinuclear distribution of rafts (arrows). Fenestrations are not resolved within the sieve plates (*) with TIRFM. (I) TIRFM of LSEC stained with Bodipy FL C5 ganglioside GM1, a marker for rafts, and CellMask Orange (orange) confirming mostly perinuclear distribution of rafts. Scale bar 5 µm (A, B, H, I), 1 µm (C–G).

To study the morphology and relationship between sieve plates and membrane rafts, we performed 3D-SIM on isolated LSECs. Figure 1 (A–G) shows representative 3D-SIM micrographs of sieve plates and membrane rafts in LSECs while Videos S1, S2, S3 demonstrate LSECs rotating in 3D space. The membrane rafts, which are stained with Bodipy FL C5 ganglioside GM1, are approximately 100 nm in size, occupy about one quarter of the surface area of the cell membrane, and protrude slightly from the cell membrane surface. They are distributed preferentially towards the perinuclear region where the rafts are aggregated in a large perinuclear ring. Several clustered rafts are apparent in the peripheral regions of the cell membrane. These are 1–2 µm in diameter, circular and have a more distinct morphology including a raised perimeter.

The fenestrations and sieve plates are also well resolved by 3D-SIM. The fenestrations are approximately 50–150 nm in diameter and are mostly found in sieve plates containing about 10–100 fenestrations. The sieve plates are more frequent in the peripheral regions of the cell. There is an inverse relationship between the distribution of sieve plates and membrane rafts.

In order to confirm the distribution of membrane rafts in LSECs and their relationship with sieve plates we also performed total internal reflectance fluorescence microscopy (TIRFM) using two rafts stains, Bodipy FL C5 ganglioside GM1 and NBD-cholesterol. Representative micrographs are shown in Figure 1 (H–I). Although TIRFM cannot visualize structures below the limit of resolution of light microscopy such as individual fenestrations, the distribution of the fluorescent raft stains confirmed the findings of 3D-SIM – rafts are preferentially distributed in the perinuclear regions of LSECs and are inversely distributed with respect to sieve plates.

### Effects of Manipulating Membrane Rafts on Fenestrations

**Figure 2:**
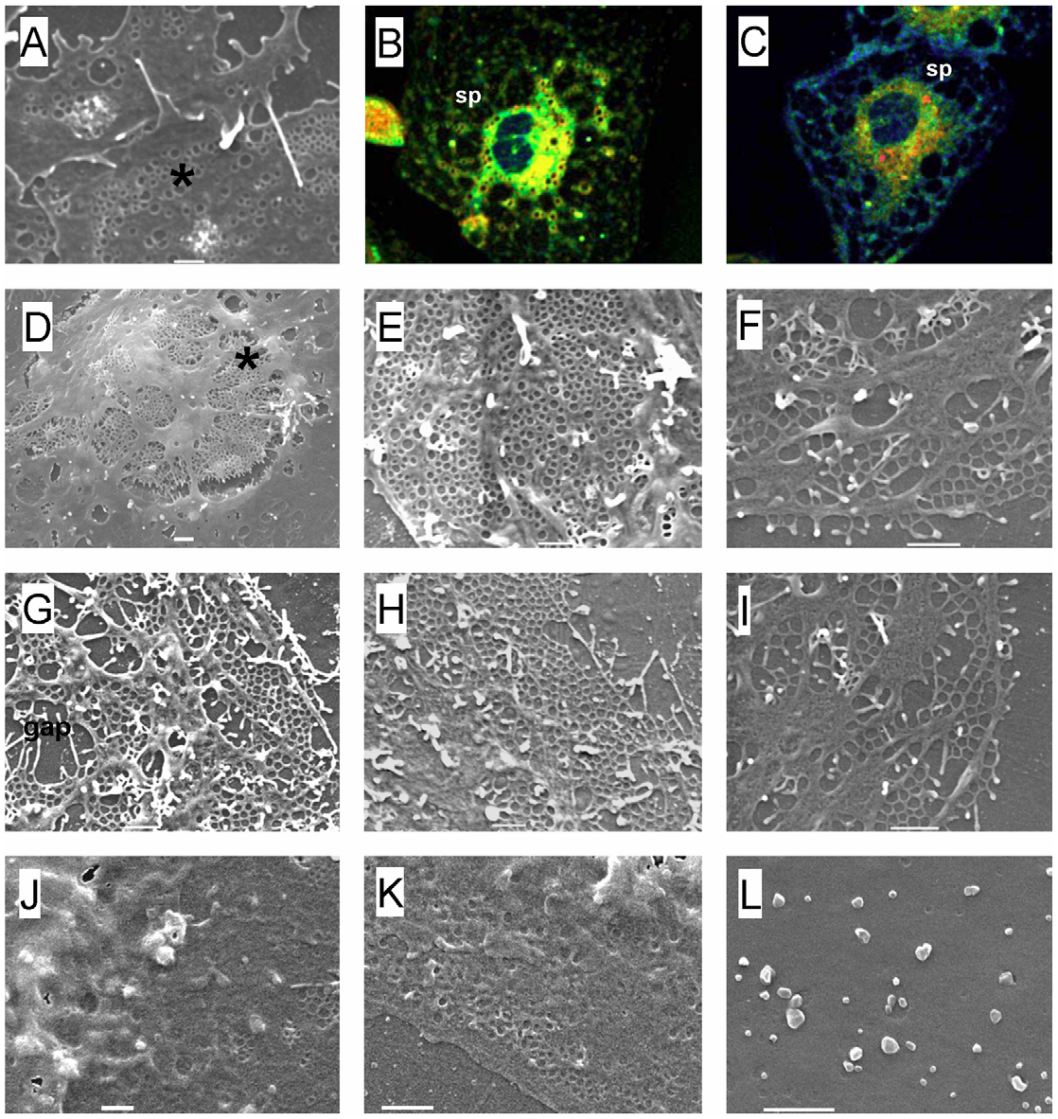
Effects of manipulating membrane rafts on fenestrations. (A) SEM of normal LSEC. Fenestrations (*) are clustered in sieve plates. (B) Two-photon fluorescence microscopy of normal LSEC stained with LAURDAN, a stain which changes from red/yellow in raft regions to blue/green in non-raft regions. There is widespread red and yellow staining consistent with membrane rafts in the cell membrane between the sieve plates (sp). Fenestrations cannot be resolved with two-photon fluorescence microscopy. (C) Two-photon fluorescence microscopy of LSEC stained with LAURDAN following treatment with 7KC. There is increased blue staining consistent with increased lipid-disordered, non-raft regions. (D–I ) SEMs of LSEC following treatment with 7KC which depletes rafts by inducing lipid disorder. There is an increased number of fenestrations and gaps are visible presumably where rafts have been disrupted, including the perinuclear region (*). (J,K,L) SEM of LSEC after treatment with Triton X-100. There is a marked decrease in fenestrations. (scale bar = 1 µM).

**Figure 3:**
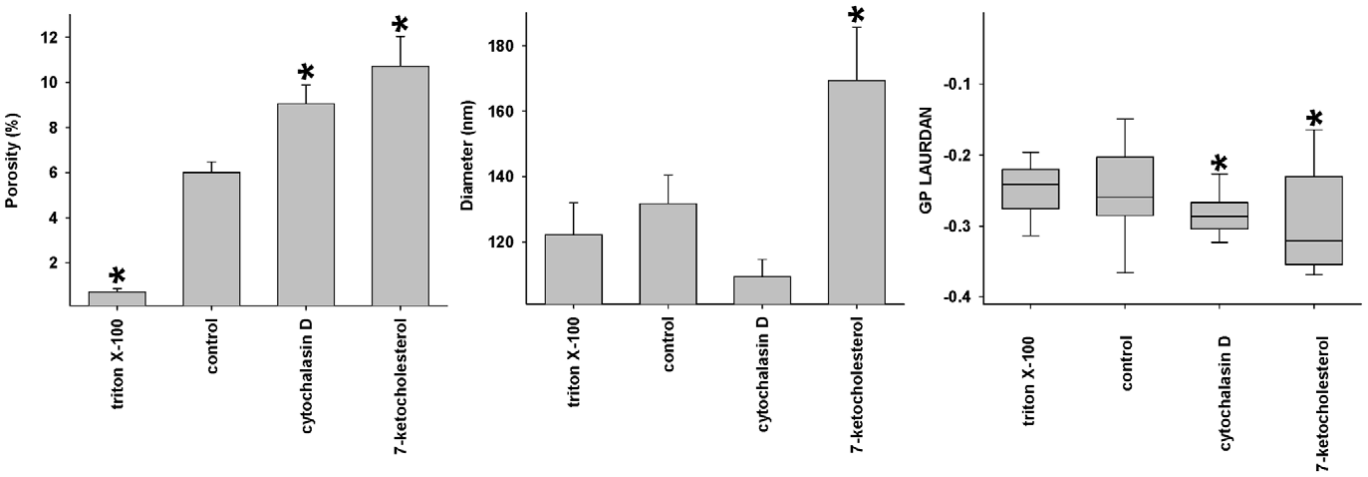
Effects of manipulating rafts on porosity. The effects of 7KC, Triton X-100 and cytochalasin D on the porosity and diameter of fenestrations (determined using SEM) and generalized polarization (GP, following staining with LAURDAN imaged using two-photon microscopy) (* significantly different from control values P<0.05, each data point represents average ± SEM of 7–28 images and 83–2840 fenestrations).

We used low concentrations of 7-ketocholesterol (7KC) in an attempt to reduce lipid-ordered membrane rafts [16]. We used low concentrations of Triton X-100 in an attempt to reduce non-raft lipid-disordered membranes [17]. Concentration-dependent effects on the morphology of LSECs of both agents are shown in Figures S1 and S2. At high concentrations, both agents cause extensive cell damage. Representative figures from the low concentration experiments are shown in Figure 2 and quantification of the results in Figure 3. We applied Triton X-100 to cells at 37°C rather than 4°C to maintain cell viability and preliminary experiments showed that this was associated with more effects on fenestrations (Figure S1). Fenestrations were quantified by measuring their diameter or their porosity (which is the percentage of the surface area of the cell membrane containing fenestrations). The effects of 7KC were initially studied at four concentrations. At the lowest concentration (9 µM) there was an increase in fenestrations while at the highest concentration (73 µM) typically used to study rafts in lymphocytes, cell membrane retraction and damage were apparent, however remaining fragments of cell membrane were highly fenestrated (Figure S2). Therefore the 9 µM concentration was used in all subsequent experiments. LAURDAN (6-lauroyl-2-dimethylaminonaphthalene) was used to visualize rafts with ratiometric two-photon fluorescence microscopy. LAURDAN undergoes a spectral shift when in lipid-disordered non-raft regions versus lipid-ordered raft domains, thus can identify both raft and non-raft regions simultaneously and is a widely accepted method for identifying lipid-disordered and lipid-ordered regions of cell membranes [18], [19]. This is quantified with Generalized Polarization values (GP) which range from −1 to 1, where −1 represents most lipid disordered and 1 represents the most lipid ordered membranes [18]. LAURDAN-stained LSECs confirmed that 7KC increased lipid disordered, non-raft regions in the cell membrane. The GP values decreased from −0.259 in controls to −0.320 in 7KC-treated cells. These results were confirmed using NBD-cholesterol where it was found that Triton X-100 increased raft staining while 7KC reduced staining (Figure S3). Scanning electron microscopy (SEM) revealed that 7KC caused a marked increase in both the diameter of fenestrations and total porosity in isolated LSECs (Figure 2 and 3). 7KC was associated with the development of some gaps which presumably represent deficits in the cell membrane where the rafts have been depleted. On the other hand, Triton X-100 was associated with a marked decrease in fenestration porosity as assessed by SEM (Figure 2 and 3). GP values did not statistically significantly change following treatment with Triton X-100 however the distribution was more homogeneous and fewer sieve plates were apparent.

### Effects of Manipulating Actin on Fenestrations

**Figure 4:**
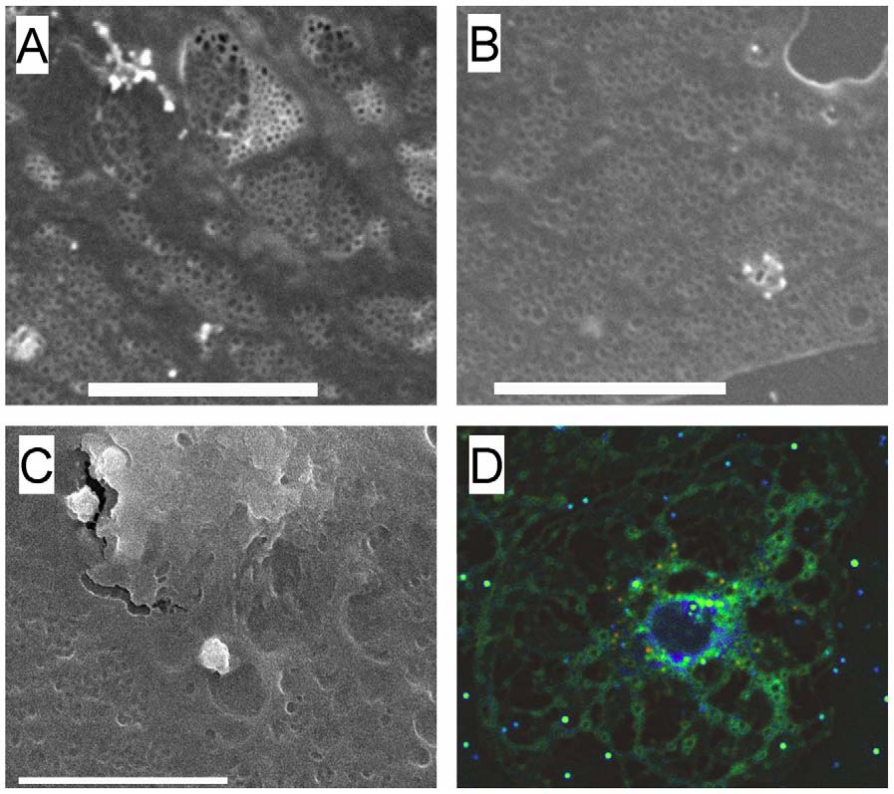
Effects of manipulating actin on fenestrations. (A) SEM of control LSEC showing fenestrations clustered in sieve plates. (B) SEM of LSEC following treatment with cytochalasin D showing an increase in fenestrations. (C) SEM of LSEC following treatment with Triton X-100 and cytochalasin D. Triton X-100 ameliorated the effects of cytochalasin D on fenestrations. (D) Two-photon fluorescence microscopy of LSEC following treatment with cytochalasin D and stained with LAURDAN. Numerous sieve plates are apparent. (scale bar 5 µM).

Actin influences both membrane rafts [13] and fenestrations [6],[7], therefore the effect of the actin-disrupting agent, cytochalasin D [20] was studied. Representative micrographs are shown in Figure 4. SEM showed that cytochalasin D increased number of fenestrations. The effect of cytochalasin D on LAURDAN staining in LSECs was studied using two-photon fluorescence microscopy. It was associated with more sieve plates and an increase in lipid-disordered staining. Then it was determined whether the effect of cytochalasin D to increase fenestrations could be abrogated by pretreating the cells with Triton X-100 to reduce non-raft regions of the membrane. We found no increase in fenestrations following co-treatment with Triton X-100 and cytochalasin D, in fact porosity was reduced substantially (porosity 0.87±0.22% following dual treatment vs 9.05±0.84% following treatment with only cytochalasin D). The results indicate that actin influences fenestrations via its effects on membrane rafts. Finally we assessed the effects of 7KC with cytochalasin D on fenestrations. As expected there was an increase in fenestrations which indicates that the disruption of rafts and actin both act via a linked or similar mechanism to increase fenestrations.

### The Presence of Membrane Pores Adjacent to Sieve Plates

**Figure 5:**
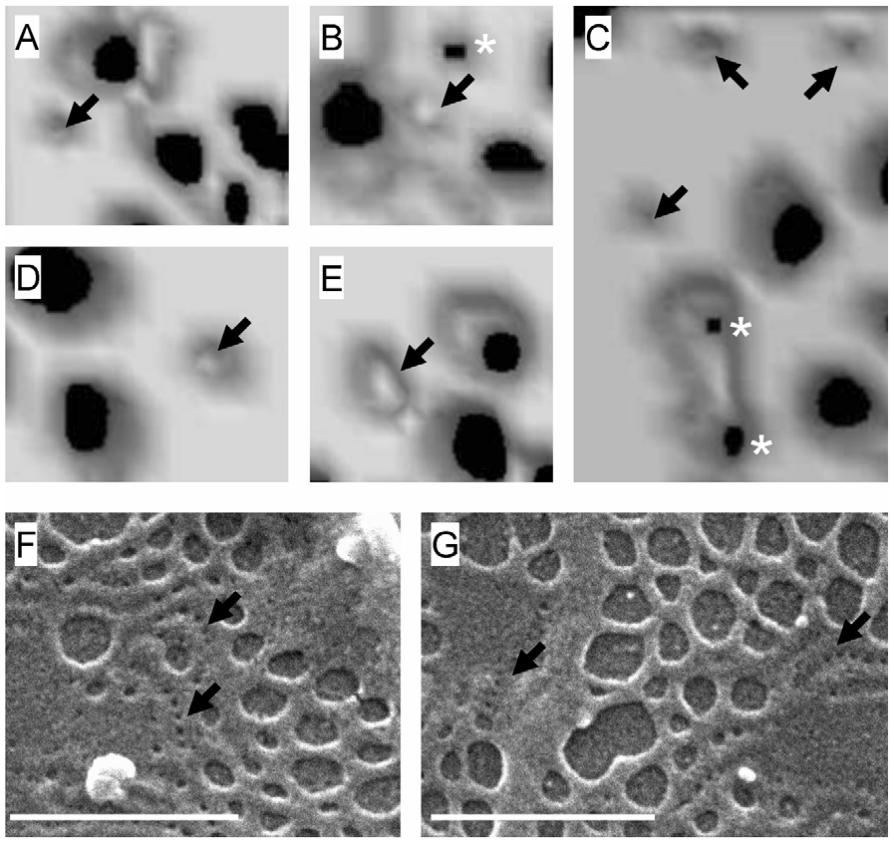
Pores and fenestrations. (A–E) Isorendered 3D-SIM reconstructions of fenestrations from LSECs. Around the sieve plates are a few pores (→) and some early fenestrations can be identified forming at the base of the pores (*). (F) Scanning electron microscopy isolated LSECs. Pores (→) similar to those seen on 3D-SIM are present towards the periphery of the sieve plates. (scale bar 1 µm).

It has been reported that vesicles form in cell membranes when the membrane-stabilizing effects of actin and rafts are depleted [21]. Therefore we examined iso-rendered 3D-SIM micrographs and SEM of fenestrations to determine whether there was any evidence for the formation of fenestrations from pores or vesicles. Pores including ones with small fenestrations at their base were apparent adjacent to the sieve plates on both 3D-SIM and SEM (Figure 5).

### Effects of VEGF on Membrane Rafts in LSECs

**Figure 6:**
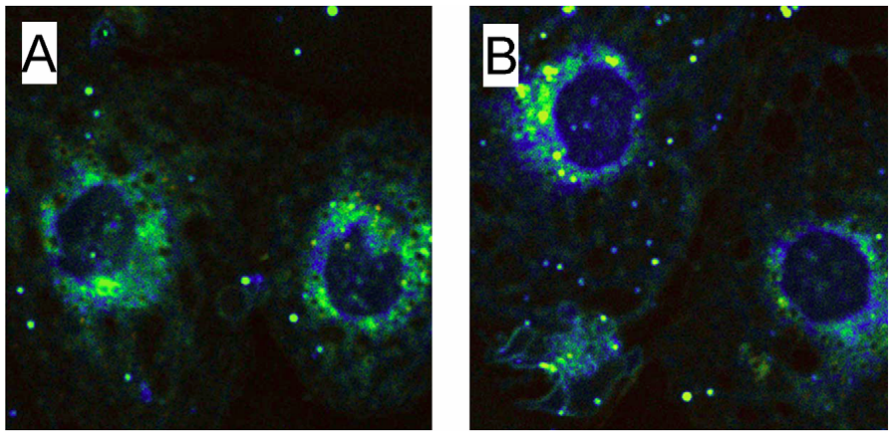
Effects of VEGF on membrane rafts. (A) Two-photon fluorescence microscopy of LSECs stained with LAURDAN. (B) Two-photon fluorescence microscopy of LSECs stained with LAURDAN following treatment with VEGF, showing increased blue staining consistent with increased non-raft lipid disordered membrane.

VEGF increases fenestrations in LSECs [8], [9]. Therefore, the effects of VEGF on fenestrations and membrane rafts in LSECs were studied (Figure 6). LSECs were stained with LAURDAN and studied using two-photon fluorescence microscopy. VEGF was associated with increased blue staining indicating an increase in non-raft, lipid disordered regions of the cell membrane (median −0.365 vs −0.259 in controls, P<0.001).

### Effect of 7KC on LSECs in the Perfused Liver

**Figure 7:**
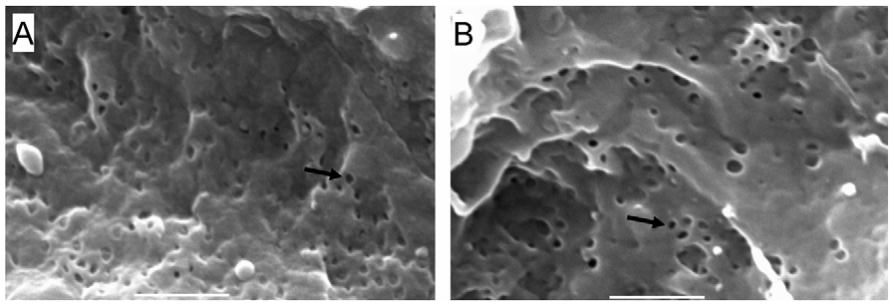
Effects of 7KC on the perfused mouse liver. (A) Scanning electron micrograph of a mouse perfused liver sinusoid. (B) Scanning electron micrograph of a mouse liver sinusoid following perfusion with 7KC (scale bar 1 µM, → fenestration).

In light of these *in vitro* results indicating that 7KC manipulates fenestrations via its effects on rafts, we tested the effect of perfusing intact livers with 9 µM of 7KC for 8 minutes (Figure 7). Treatment with 7KC caused ruffling of LSEC membranes and increased the diameter of fenestrations from 80.9±0.8 nm in perfused untreated control mice to 85.8±1.0 nm in those perfused with 7KC (P<0.001). However porosity of the LSEC was unaffected by treatment with 7KC (7KC 1.79±0.25% vs control 1.72±0.17%).

## Discussion

Fenestrations and rafts are both cell membrane structures that are below the limit of resolution of light microscopy [1]. The morphology of fenestrations has been studied primarily using electron microscopy while that of rafts has been studied using fluorescence microscopy [15]. 3D-SIM is a super-resolution fluorescence microscopy technique that provides the opportunity to simultaneously study both membrane rafts and fenestrations and their distribution in isolated LSECs. 3D-SIM provides high resolution images of fenestrations and associated structures, such as the cellular cytoskeleton [11]. Here, we also applied 3D-SIM to visualize membrane rafts. Using the fluorescent raft stain, Bodipy FL C5 ganglioside GM1, membrane rafts were found to be aggregated preferentially in the perinuclear region of LSECs, with a more diffuse distribution in the peripheral cytoplasmic extensions, and were generally thicker than the surrounding cell membrane. This pattern of distribution of rafts was confirmed using TIRFM with two raft stains, Bodipy FL C5 ganglioside GM1 and NBD-cholesterol. With 3D-SIM, a few clustered rafts sections were also apparent in the peripheral regions of the cells. These were about 1–2 µm in diameter and some had a raised perimeter, consistent with predictions based on line tension [22]. As reported previously [11], 3D-SIM revealed that fenestrations are clustered in groups of 10–100 fenestrations called liver sieve plates that occupy 5–10% of the entire cell membrane. Moreover, the SIM images revealed that there is a distinct inverse distribution between liver sieve plates and membrane rafts. On the basis of this observation, we investigated whether manipulating membrane rafts had any effect on fenestrations.

Most studies of membrane rafts have used various agents to isolate raft or non-raft membranes. At low concentrations, 7KC disrupts membrane rafts by disordering lipid membranes [16], [19], while at much higher concentrations than we used 7KC can also induce apoptosis [23]. Triton X-100 is a detergent that has been used to separate detergent-resistant membranes from cells [17]. This has usually been undertaken using high concentrations above the Critical Micelle Concentration which are associated with cell lysis. In our experiments we used a much lower concentration in order to study cell membranes that have remained intact. Triton X-100 still penetrates cell membranes in the monomeric form [24] and also increases the formation of rafts [25]. Here we used these two agents to assess their effect on the morphology of the LSEC cell membrane. Treatment with 7KC was associated with increased number and diameter of fenestrations *in vitro* and increased diameter of fenestrations i*n vivo.* Triton X-100 was associated with a reduced number of fenestrations. The fluorescent stains, LAURDAN and NBD-cholesterol confirmed an effect of low concentrations of 7KC and Triton X-100 on membrane rafts. The inverse distribution of rafts and sieve plates observed with 3D-SIM combined with the effects of Triton X-100 and 7KC on fenestrations, suggest that rafts prevent the formation of fenestrations and sieve plates in LSECs.

To study this further, we then investigated the effects of the actin disrupter, cytochalasin D. The actin cytoskeleton is bound to membrane rafts via a range of proteins and this serves to tether and maintain raft structure [13], [14]. Cytochalasin D has been shown to disrupt membrane rafts through its effects on the actin cytoskeleton [26]. Furthermore, cytochalasin D and other actin disrupters have been reported to increase fenestrations [6], [7]. Indeed we found that cytochalasin D reduced rafts in LSECs and that this was associated with increased fenestrations. However, the effect of cytochalasin D on fenestrations was blocked and reversed by Triton X-100. On the other hand the effects of 7KC and cytochalasin D were possibly synergistic in increasing fenestrations. Although this is most likely to indicate the addition of two sub-maximal responses, it is not inconsistent with two separate mechanisms.

The results of these experiments suggest that actin and rafts reduce the formation of fenestrations in LSECs, and that fenestrations form in non-raft regions of the LSEC membrane. Disruption of actin increases fenestrations through its effect on rafts and can be prevented by the removal of non-raft regions. The processes leading to the formation of fenestrations may be similar to the generation of membrane vesicles. It has been reported that disruption of actin cytoskeleton associated with increased lipid-disordered, non-raft membrane is required for the formation of microvesicles [21]. Vesiculation occurred spontaneously in membranes when line tension associated with rafts was reduced and the tethering by actin cytoskeleton released. We were able to identify small pores in the non-raft regions of the LSEC and adjacent to the sieve plates that might represent early development of fenestrations, although it is not possible to determine what happens to these pores over time. Thus here we propose that a sieve-raft theory to explain the formation of fenestrations. Fenestrations form in non-raft membranes once the stabilizing effects of actin and rafts are depleted. Because the cytoplasmic extensions of LSECs are very thin (approximately 100 nm thick), fenestrations form rather than vesicles. The fact that small pores can develop spontaneously in lipid bilayers under appropriate conditions has been established [27], [28] and sieve-like sets of small pores generate increased stability for rafts [27].

Other pathways have been reported to be involved in the regulation and formation of fenestrations. Most of these are consistent with the sieve-raft theory because they act via the actin cytoskeleton, such as serotonin [4], VEGF [9] and rho [29]. There has also been a report that fenestrations might be a type of caveolae [30], [31] which would suggest that they reside in rafts, rather than non-rafts. However we believe that this is unlikely because caveolin-1 knockout mice have normal fenestrations [32]. To test whether VEGF exerts its action on fenestrations via rafts, we studied the effects of VEGF on LAURDAN staining in isolated LSECs. VEGF was associated with an increase in lipid-disordered membranes, consistent with this hypothesis.

In conclusion 3D-SIM revealed the three dimensional morphology of fenestrations and rafts and identified their inverse distribution in LSECs. The experiments reported here are consistent with a sieve-raft interaction, where fenestrations form in non-raft regions of LSECs once the membrane-stabilizing effects of actin cytoskeleton and membrane rafts are diminished. Agents that regulate fenestrations might act via their effects on actin and rafts.

## Materials and Methods

### Animals

3–4 and 12 month old C57/Bl6 mice were obtained from the Animal Resource Centre in Perth Western Australia. Animals were housed at the ANZAC Research Institute on a 12 hour light/dark cycle and provided with *ad libitum* access to food and water. The study was approved by the Animal Welfare Committee of the Sydney South Western Area Health Service.

### Materials

Reagents included: Liberase TM Research Grade (Roche, Basel, Switzerland); RPMI (Gibco Grand Island, NY), Percoll, Cytochalasin D, Triton X-100, methyl-β-cyclodextrin, 7-ketocholesterol, mouse recombinant vascular endothelial growth factor VEGF (Sigma Aldrich, St Louis, MO). Stains included 6-lauroyl-2-dimethylaminonaphthalene LAURDAN, Cell-Mask Orange, Bodipy FL C5 ganglioside GM1 and NBD-cholesterol (Invitrogen, Eugene, OR).

### LSEC Isolation

Mouse LSEC isolation was performed as described previously [33] by perfusion of the liver with Liberase TM (0.15 Wünsch units/ml). Non-parenchymal cells were removed by a two-step Percoll gradient and Kupffer cells were removed by selective adherence to plastic. LSECs (seeded at 0.5×10^6^ cells/cm^2^) were cultured (37°C, 5% CO_2_) in serum free RPMI-1640 for 3 hours before use.

### LSEC Treatments

Cells were treated with a variety of agents and probes to elucidate the relationship between rafts, actin and fenestrations. Membrane rafts were disrupted using 7KC [16] while non-raft regions were removed using Triton X-100 [17]. Actin was disrupted using cytochalasin D [20]. All experiments were performed in triplicate. 7KC stock solution was prepared by drop-wise adding 15 mg/ml 7KC solution in ethanol to 50 mg/ml methyl-β-cyclodextrin in PBS at 80°C to a final sterol concentration of 1.5 mg/ml. 5, 10 or 20 µl of this solution were then added to 1 ml of cell medium to obtain 9, 18, 36 or 73 µM 7KC concentrations respectively. LSECs were treated for 7 min. For Triton X-100 experiments, LSECs were incubated with 0.001% Triton X-100 in RPMI for 1 minute. For cytochalasin D experiments, LSECs were incubated with 0.5 µg/ml cytochalasin D in RPMI for 30 minutes. Experiments were also performed with both cytochalasin D with Triton X-100, and cytochalasin D with 7KC. In addition, experiments were performed where LSECs were incubated with VEGF (100 ng/ml) for 4 hours.

### Liver Perfusion with 7KC

Liver perfusions were performed in 12 month old mice as previously described [34]. The perfusate was Krebs-Henseleit bicarbonate buffer (10 mmol/L glucose, *p*H 7.4, saturated with 95% O_2_/5% CO_2_, 1% bovine serum albumin, 37°C). The perfusate flow rate was maintained at approximately 2 mL/min/g of liver using a cartridge pump (Masterflex L/S, model 794-32; Cole-Palmer, Extech Equipment, Boronia, Australia) in a non-recirculating system. Viability was confirmed by macroscopic appearance, portal venous pressure, light microscopy and electron microscopy. Control animals (n = 3) were perfused with Krebs Henseleit buffer for 10 min and 7KC treatment animals (n = 3) were perfused for 2 min with Krebs Henseleit buffer alone followed by 8 min with 9 µM 7KC solution in Krebs Henseleit buffer. After completion of treatment experiments, liver specimens were fixed for electron microscopy by gravity-fed perfusion with 2% glutaraldehyde/3% paraformaldehyde in 0.1 mol/L sodium cacodylate buffer (0.1 mol/L sucrose, 2 mmol/L CaCl_2_). Randomly selected specimens were analysed by SEM as described below.

### Fixation, Staining and Imaging of LSECs

SEM was performed as described [35], [36]. Isolated LSECs were fixed in 2.5% glutaraldehyde in 0.1 mol/L sodium cacodylate buffer, osmicated, dehydrated in ethanol and hexamethyl-disilazane, mounted on stubs, sputter coated with platinum, and examined using a JEOL 6380 Scanning Electron Microscope. Figures at 10,000× magnification were used to measure fenestration diameter and LSEC porosity using Image J (http://rsb.info.nih.gov/ij/, between 464–2483 fenestrations assessed in each treatment group). Porosity is defined as the percentage of cell membrane covered by fenestrations.

3D-SIM was performed as described previously [11]. LSECs were stained with Cell-Mask Orange (Life Technologies, Carlsbad, CA) which is a cell membrane marker, and Bodipy FL C5 ganglioside GM1 which is a marker of membrane rafts then fixed with 4% fresh paraformaldehyde in PBS. The cells were imaged with a structured illumination microscope based on the Deltavision/OMX V2.0 (Applied Precision Inc, Issaquah, WA). Image reconstructions were made with the OMX specific SoftWoRx v4.5.0 software package (Applied Precision Inc, Issaquah, WA). Three dimensional figures were generated by iso-surface rendering (iso-rendering) which builds up a 3D model from multiple two dimensional images (Volocity 3D Image Analysis Software, PerkinElmer, MA).

TIRFM was performed as described previously [18] using Bodipy FL C5 ganglioside GM1 or NBD-cholesterol. A custom built microscope was used with excitation at 473 nm from a diode-pumped solid-state laser delivered via a single mode optical fibre and a rotatable mirror. Excitation was delivered into the backport of an inverted epifluorescence IX71 Olympus microscope equipped with a 60×1.45 NA oil-immersion TIRF objective. Fluorescence was collected on an electron-multiplying CCD camera in the range 500–593 nm and 600–680 nm using a two-channel imager.

Two-photon fluorescence microscopy was performed as described [18]. LAURDAN undergoes a spectral blue-shift from 490 nm when in lipid-disordered non-raft regions to 440 nm when in lipid-ordered raft domains, thus can identify both raft and non-raft regions simultaneously [18]. Two-channel time-resolved live cell imaging was performed using a confocal laser-scanning microscope (TCS SP5, Leica Microsystems GmbH, Wetzlar, Germany) with a 1.2NA 63× water-immersion objective and multiphoton excitation from a mode-locked, femtosecond-pulsed Ti:Sapphire laser (Mai-Tai, Spectra Physics, Mountain View, CA). LAURDAN was excited at 800 nm and fluorescence was split using a dichroic mirror (458 nm) passed through a bandpass filter centered on 425 and 483 nm. Fluorescence was quantified using ImageJ and converted to generalized polarization (GP) values (n = 30–143 cells for each group) [18].

### Statistics

Results are presented as mean ± SEM or median. Multiple groups were compared with either ANOVA with a post-hoc Student-Newman-Keuls test, or Kruskal-Wallis test with a post hoc Dunn’s method (Sigmastat v11, Systat Software Inc).

## Supporting Information

Figure S1 Concentration-dependent effects of Triton X-100 on isolated LSECs. Scanning electron micrographs of LSECs after treatment with 0.1, 0.01 and 0.001% Triton X-100 at 25C. The effects of Triton X-100 were diminished when performed at 4C, while cell damage occurred with higher concentrations of Triton X-100. (scale bar 1 µm)
(TIFF)

Figure S2 Concentration-dependent effects of 7KC on isolated LSECs. Scanning electron micrographs of LSECs after treatment with 18, 36 and 73 µM 7KC. Cell damage occurred at higher concentrations of 7KC. (scale bar 1 µm)
(TIFF)

Figure S3 The effects of 7KC (9 µM) and Triton X-100 (0.0001%) on NBD-cholesterol staining in isolated LSECs. There was an increase in staining with Triton X-100 and a reduction with 7KC.
(TIFF)

Video S1 3D-SIM of LSECs stained with Bodipy FL C5 ganglioside GM1, a marker for rafts (green) and Cell-Mask Orange, a cell membrane marker (orange). There is an inverse distribution between liver sieve plates and membrane rafts.
(MOV)

Video S2 3D-SIM of LSECs stained with Bodipy FL C5 ganglioside GM1, a marker for rafts (green) and Cell-Mask Orange, a cell membrane marker (orange). There is an inverse distribution between liver sieve plates and membrane rafts.
(MOV)

Video S3 3D-SIM of LSECs stained with Bodipy FL C5 ganglioside GM1, a marker for rafts (green) and Cell-Mask Orange, a cell membrane marker (orange). There is an inverse distribution between liver sieve plates and membrane rafts.
(MOV)
