# Supplementary figures and images for: The Relationship between Fenestrations, Sieve Plates and Rafts in Liver Sinusoidal Endothelial Cells
Source: PLoS One. 2012 Sep 24;7(9):e46134. doi: 10.1371/journal.pone.0046134 (PMC3454341; doi:10.1371/journal.pone.0046134)

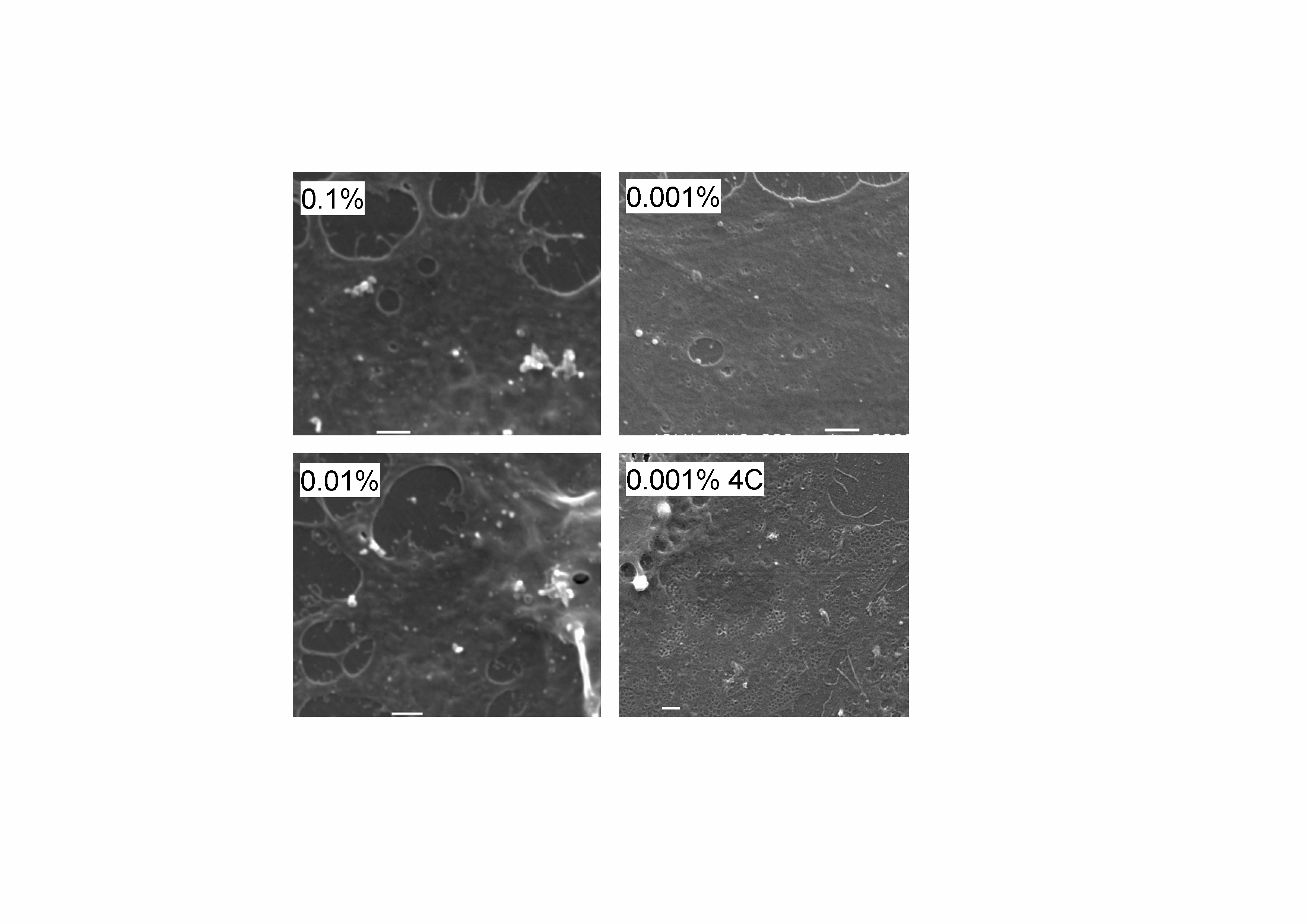

Supplement: Figure S1 — Concentration-dependent effects of Triton X-100 on isolated LSECs. Scanning electron micrographs of LSECs after treatment with 0.1, 0.01 and 0.001% Triton X-100 at 25C. The effects of Triton X-100 were diminished when performed at 4C, while cell damage occurred with higher concentrations of Triton X-100. (scale bar 1 µm) (TIFF) [file pone.0046134.s001.tiff]

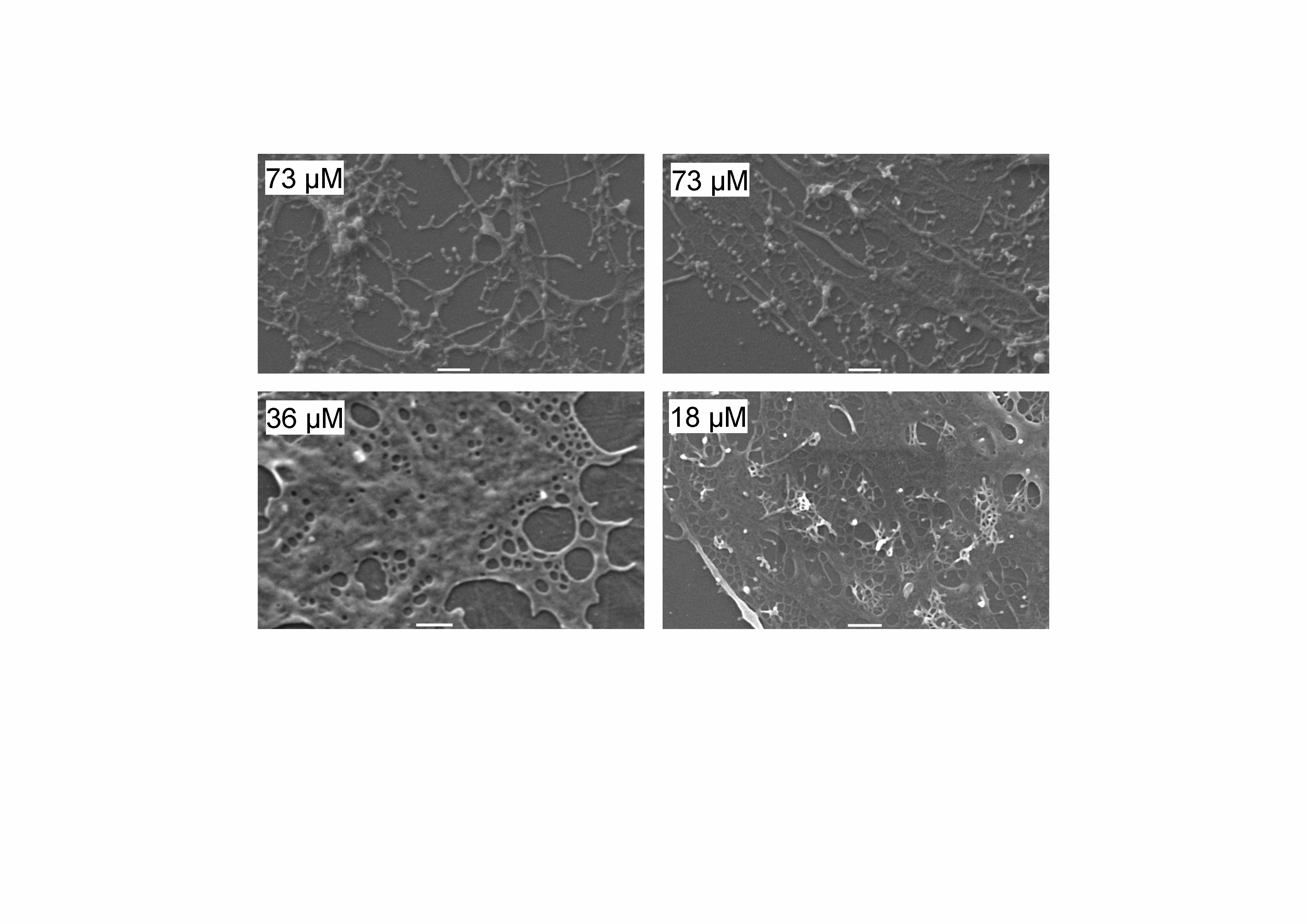

Supplement: Figure S2 — Concentration-dependent effects of 7KC on isolated LSECs. Scanning electron micrographs of LSECs after treatment with 18, 36 and 73 µM 7KC. Cell damage occurred at higher concentrations of 7KC. (scale bar 1 µm) (TIFF) [file pone.0046134.s002.tiff]

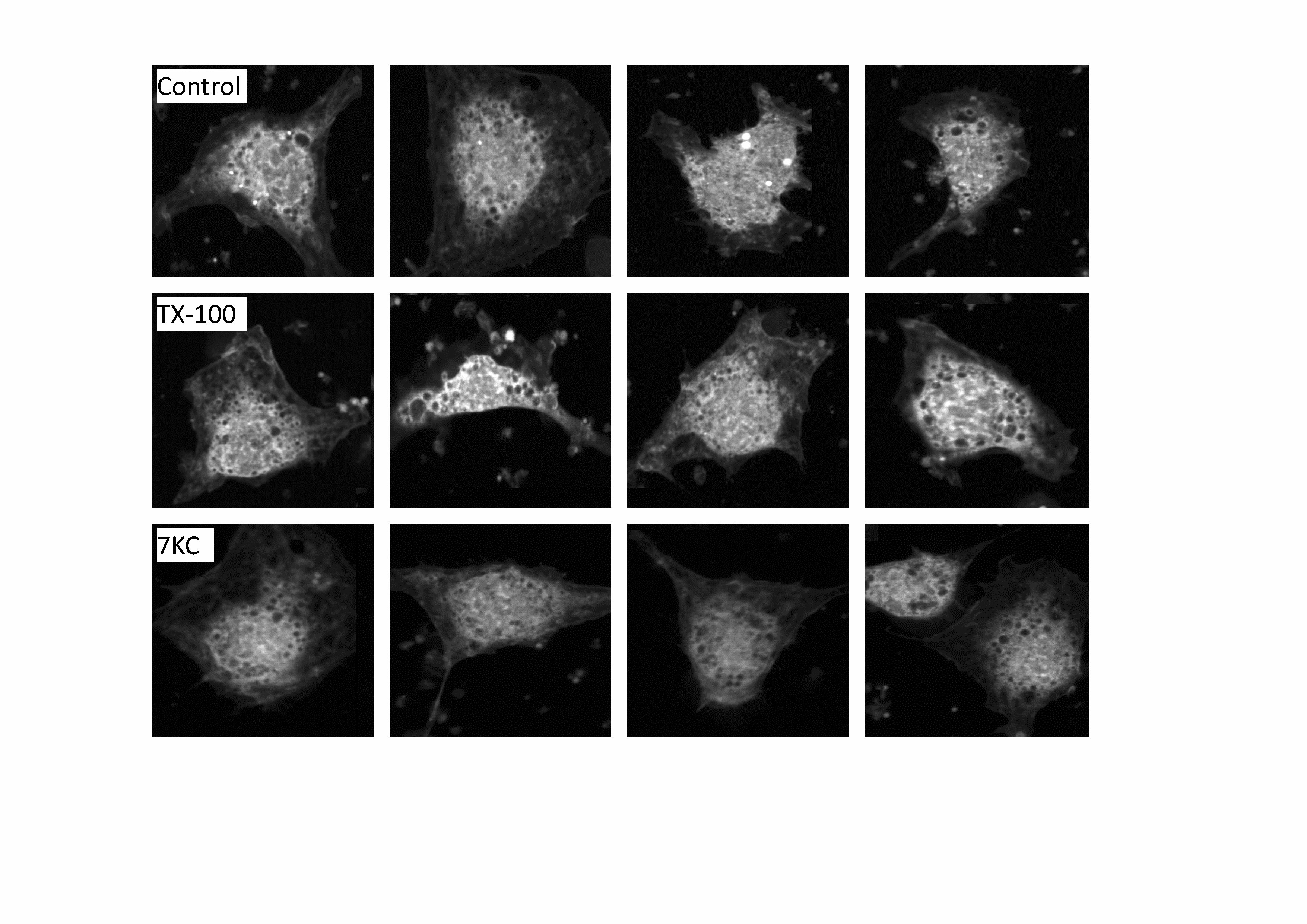

Supplement: Figure S3 — The effects of 7KC (9 µM) and Triton X-100 (0.0001%) on NBD-cholesterol staining in isolated LSECs. There was an increase in staining with Triton X-100 and a reduction with 7KC. (TIFF) [file pone.0046134.s003.tiff]
